# *Enterotoxigenic Escherichia coli* Interferes FATP4-Dependent Long-Chain Fatty Acid Uptake of Intestinal Epithelial Enterocytes via Phosphorylation of ERK1/2-PPARγ Pathway

**DOI:** 10.3389/fphys.2019.00798

**Published:** 2019-06-20

**Authors:** Zhi Li, Heyuan Liu, Bocheng Xu, Yizhen Wang

**Affiliations:** National Engineering Laboratory of Biological Feed Safety and Pollution Prevention and Control, Key Laboratory of Animal Nutrition and Feed, Ministry of Agriculture, Key Laboratory of Animal Nutrition and Feed Science of Zhejiang Province, Zhejiang University, Hangzhou, China

**Keywords:** CD36, ERK1/2, *Enterotoxigenic Escherichia coli*, fatty acid uptake, FATP4, PPARγ, phosphorylation

## Abstract

Sufficient fatty acid (FA) uptake from jejunal lumen is closely associated with pediatric growth. *Enterotoxigenic Escherichia coli* (ETEC), which poses a big threat to young mammals’ health, is also targeted on the jejunum, however, the effects on FA uptake is not understood yet. To explore the impacts of ETEC on the FA uptake ability of jejunum epithelial enterocytes during early life, we orally gavaged weaning piglets with ETEC K88 and found intestinal inflammation combined with compromised uptake of LCFA (C16:0, C18:0, C20:3, C20:4) except for C14:0 whose chain length is similar to medium chain fatty acid (MCFA). Furthermore, we observed reduced protein expression of TJs, fatty acid transport protein 4 (FATP4), peroxisome proliferator-activated receptor γ (PPARγ), phosphorylated extracellular signal-regulated kinase 1/2 (p-ERK1/2), and upregulated expression of p-PPARγ. In the *in vitro* study, we challenged polarized porcine intestine jejunum cell line IPEC-J2 with ETEC K88 and discovered similar results on intestinal barrier and expression of associated genes combined with morphological changes. Based on the constructed cellular model, we then determined lower uptake of BODIPY-labeled C16:0 without any difference in the uptake of BODIPY-labeled C12:0. The content of intracellular triglyceride which was mainly synthesized by LCFA concomitantly lowered down. Using gene knock down and overexpression, FATP4 was confirmed to be responsible for LCFA uptake. Moreover, ERK1/2 inhibitor U0126 and PPARγ antagonist T0070907 revealed ETEC could initiate cascaded phosphorylation of ERK1/2 and PPARγ resulting in hindered expression of FATP4. These results indicate ETEC challenge will cause dysfunction in FATP4-dependent LCFA uptake by phosphorylation of ERK1/2 and PPARγ. Furthermore, intestinal uptake of MCFA is in a FATP4-independent manner which is not easily disturbed by ETEC.

## Introduction

Sufficient FA uptake is of great importance for the health of human beings. On the first hand, FA provides around 30% of total energy for adults (Willett et al., 2019). On the other hand FA participates in biomembrane constitution, receptor activation, signal transduction, neurological development and the regulation of various genes (Fritsche, 2006; Glatz and Luiken, 2017). For young children FA uptake plays a more critical role (Willett et al., 2019). Insufficient FA supply not only exacerbates the children physical conditions but also has a profound impact on the entire life (Alles et al., 2014).

Due to the vital properties FA uptake mechanism has already been extensively revealed, whereas how it reflects to microbial infection is not fully elucidated yet. ETEC, one of the major intestinal pathogenic bacteria, is capable of paralyzing intestinal barrier, causing watery diarrhea or even death especially on young mammals (Croxen and Finlay, 2010; McLamb et al., 2013; Adewole et al., 2016). Because of the overlap of adhesion and FA uptake site (Caspary, 1992; Mariadason et al., 2005), ETEC infection is putative to result in the change of FA uptake through alteration of transporting proteins. Among the reported proteins in small intestine, CD36 and FATP4 are widely acknowledged as the most relevant transporters and therefore may be the targets for ETEC (Pohl et al., 2005; Nickerson et al., 2009; Abumrad and Goldberg, 2016; Glatz et al., 2016; Kim and Dyck, 2016). We thereby speculated ETEC may influence the expression of CD36 and/or FATP4 by which interfered the FA uptake in small intestine.

What participates in the regulation of FA uptake proteins still remains obscure. According to our literature summary, PPARγ and ERK1/2 have been reported to be located in the upstream of FA uptake. The effects of PPARγ on intestinal FA absorption through regulation of CD36 and/or FATP4 have been revealed in a series of *in vivo* and *in vitro* studies, whereas most of them were not conducted on intestinal enterocytes or tissues (Schaiff et al., 2007; Kanda et al., 2009; Goto et al., 2013; Briot et al., 2018). Upon ETEC infection ERK1/2 can be phosphorylated by lipopolysaccharide – a key component of ETEC cell wall – and further induces disruption of TJs as well as inflammatory responses (Gonzalez-Mariscal et al., 2008; Mizuno et al., 2011; Yu et al., 2015). Inhibition of ERK1/2 phosphorylation can maintain PPARγ in the non-phosphorylated, active state (Rangwala et al., 2003; Burns and Vanden Heuvel, 2007; Papageorgiou et al., 2007; Hosooka et al., 2008). However, in some cases PPARγ can also impact the phosphorylation of ERKs as comprehensively reviewed by Burgermeister and Seger (2008), which indicates the interaction between ERK1/2 and PPARγ still requires more investigation.

The ultimate purpose of the current study is to gain insights into the effects of ETEC on intestinal FA uptake. We postulated that ETEC would negatively impact FA uptake proteins CD36 and/or FATP4 by which disturb the FA uptake from the lumen, which is probably conducted by ERK1/2 and PPARγ signaling pathway.

## Materials and Methods

### Cultivation of ETEC

*Enterotoxigenic Escherichia coli* K88 used in this study was provided by Ph.D. candidate Xin Zong (Institute of Feed Science, Zhejiang University, China). Single colony was picked from agar plate and cultivated in Luria-Bertani (LB) broth (L8291, Solarbio, China) overnight in shaker (37°C, 150 RPM). The culture was then diluted in fresh LB broth at 1:100 and continued shaking until the optical density (600 nm) reached 0.49–0.51, which was recognized as the marker of mid-log phase and the bacteria density was 10^8^ CFU/mL using cell counting chamber under microscope. Bacteria were centrifuged (room temperature, 2,000 RPM) for 10-min and then were resuspended in phosphate buffered saline (PBS) which was repeated once. The precipitation would be suspended in PBS for piglet challenge or in antibiotic-free media for cell challenge.

### Challenge of Piglets With ETEC

Animal studies were approved by the Zhejiang University Animal Care and Use Committee. All trials were conducted under the supervision of the committee. The ration was formulated as a powder form according to the piglet nutrition requirement of National Research Council (2012).

A total of 12 healthy 28-day-old Duroc × Landrace × Yorkshire piglets (average initial weight 7.30 ± 0.12 kg) were obtained from Shaoxing Keqiang Co., Ltd., (China). Piglets were randomly divided into two groups with six duplicates of one head in each. After 4-day preliminary feeding, on day 5 and day 6 the treatment group was gavaged with 50 mL PBS containing 10^6^ CFU/mL ETEC per head and the control group was gavaged with the same volume of PBS. During day 7 to day 9, fecal consistency score was recorded to measure the state of intestinal disease according to the following criterion (Adewole et al., 2016): 0, solid feces; 1, soft feces; 2, mild diarrhea; and 3, severe diarrhea. Piglets with a fecal score >1 were considered as diarrheal. On day 10 all piglets were gavaged with olive oil (Betis Products, Spain) and after 3 h, serum and jejunum samples were collected. Serum was used for FA content quantification via gas chromatographic analysis (Yi et al., 2017) and for D-lactate determination via porcine D-lactate colorimetric assay kit (K667-100, BioVision, United States). Samples of jejunum were used for western-blot assay. Before gavage, blood collection and the final sacrifice, piglets were firstly anesthetized by isoflurane with respect to their wellbeing.

### Cultivation and Polarization of IPEC-J2 Cell Line

According to Brosnahan and Brown (2012), IPEC-J2 cells were cultivated on 0.4 μm collagen-coated Transwells (3491, Corning, United States) in DMEM/F12 media containing 2 mM L-glutamine, 15 mM HEPES (CM0403, Yocon, China), supplemented with 1% penicillin-streptomycin (67107346, Biosharp, China). The cultivation condition was 37°C, 5% CO_2_, and 90% relative humidity. Volume of media added into upper and lower chamber of Transwell was 1.5 and 2.5 mL, respectively. Each day media were replaced and TER was recorded using electrical resistance tester (MERS00002, Millipore, United States). The period for polarization was to be implied in cultivation where Transwell was not available. All of the following assays without special notes were performed on polarized IPEC-J2 cells.

### Scanning Electron Microscopy Analysis of Microvilli

For SEM analysis cells were cultivated in cover glasses (WHB-12-CS, Shanghai Wohong Biotechnology, China) within Transwell. Firstly, cells were fixed in 2.5% glutaraldehyde in PBS (0.1 M, pH 7.0) overnight and then were postfixed with 1% OsO_4_ in PBS for 1.5-h. In the following cell samples were dehydrated in a graded series of ethanol (30, 50, 70, 80, 90, and 95%) for about 15-min at each step followed by dehydration twice in alcohol (100%) for 20-min. Between the treatment of different solutions cell samples were washed three times in PBS for 15-min per time. SEM analysis of the microvilli on the cell surface was performed by ultra-high resolution scanning electron microscope (SU8010, Hitachi, Japan) in Analysis Center of Agrobiology and Environmental Sciences, Zhejiang University (China).

### Challenge of Polarized IPEC-J2 With ETEC

After the antibiotic-containing media were removed and the plate was washed softly with PBS for three times, ETEC-containing (10^6^ CFU/mL) media were added into each well. The challenge period was 3-h at the same cultivation condition. ETEC-containing media were then removed followed by three times’ wash with PBS. Afterward cells were harvested or preserved properly according to the requirements of further assays.

### Bodipy-Labeled FA Uptake Measurement and Fluorescence Confocal Microscopy

Bodipy-labeled fluorescent FA is widely used in FA uptake studies as a bio-marker due to the convenience for detection and low cytotoxicity (Niot et al., 2009; Siddiqi et al., 2010; Nakamura et al., 2014). In this study C1-Bodipy-C12 (D3822, Invitrogen, United States) and C1-Bodipy-C16 (D3821, Invitrogen, United States) were, respectively, used to measure the uptake of MCFA and LCFA. The working solution and treating method were referred to Choi et al. (2002) and Niot et al. (2009). Cells for quantification analysis were cultivated in 96-well black/clear plate (3603, Corning, United States) for polarization. The working solution was 50 μL MEM (51200-038, Gibco, United States) and 50 μL Hank’s Balanced Salt Solution (14175-095, Thermo Fisher Scientific, United States) containing 5 μM Bodipy-labeled FA, 3.9 mM trypan blue, 0.5 g/L gentamicin and 5 mM taurocholic acid sodium salt (T8510, Solarbio, China), assisting FA huddle into micelles to mimic the lumen condition. After 10-min cocultivation with working solution, the plate was read using microplate reader (SpectraMax M5, Molecular Devices, United States). The excitation wave was 485 nm and the emission wave was 528 nm. The fluorescence of each well was normalized by the fold change of cell quantity using CCK8 kit (CK04, Dojindo, Japan). Cells for confocal microscopy observation were cultivated on confocal dishes (J40141, Jing’an, China) for polarization. The working solution was 1 mL. After 10-min the working solution was softly washed with and replaced by PBS. The cells were observed using Zeiss LSM 780 confocal microscope (Zeiss, Germany) with excitation wave of 485 nm and emission wave of 528 nm. The pictures were analyzed using ZEN 2012 software (Zeiss, Germany).

### Intracellular Triglyceride Assay

As the preliminary experiment, starvation was performed to eliminate the variance in endogenous TG content based on which to compare the uptake ability in a uniform condition. Treatment of each group was as follows: (1) serum, 3.5-h cultivation in MEM media containing 10% fetal bovine serum (FBS) (900-108, Gemini, United States) and 1% gentamicin; (2) MEM, 3.5-h cultivation in MEM media without FBS; (3) MEM + serum, 3-h treatment like MEM treatment followed by 30-min treatment as serum treatment. The intracellular TG content was tested using the TG assay kit (GPO-POD, Applygen, China) according to the manufacturer’s recommended protocol accompanied with normalization by total protein content using BCA kit (A045-3, Jiancheng, China).

In the formal assay, MEM + serum treatment was used as control and ETEC treatment was cells challenged and starved simultaneously at the ETEC-containing MEM for 3-h followed by 30-min serum treatment without ETEC.

### Knock-Down and Overexpression of FATP4 in Polarized IPEC-J2 Cell Line

For knock-down assay, siRNA-FATP4 was chemically synthesized with primers as follow: sense GCGCACGGUG CCCAUCUUATT and antisense UAAGAUGGGCACC GUGCGCTT. The transfection experiment was conducted using siRNA-mate kit (GenePharma, China) under the manufacturer’s protocol. For overexpression, sequence of FATP4 was synthetized and inserted into the plasmid pEX-3. The construction was transfected into polarized IPEC-J2 cell line according to the provided protocol of GenePharma (China). All the sequences were synthesized by GenePharma (China). The impacts of knock-down and overexpression were measured by western-blot analysis of FATP4.

### Treatment of ERK1/2 Inhibitor U0126 and PPARγ Antagonist T0070907 in Polarized IPEC-J2 Cell Line

To demonstrate the involvement of ERK1/2 and PPARγ, 20 μM ERK inhibitor U0126 (MCE, China) and 5 μM PPARγ antagonist T0070907 (MCE, China) was added into polarized IPEC-J2 cells. Treatment period of U0126 and T0070907 was 2 and 24 h. The inhibition and antagonism effects were measured by western-blot analysis of non-phosphorylated and phosphorylated proteins (ERK1/2 and p-ERK1/2; PPARγ and p-PPARγ) (Zaytseva et al., 2011; Ong et al., 2015; Li et al., 2017).

### Gene Transcription Analysis by Real-Time Quantitative PCR

The real-time quantitative PCR (RT-qPCR) assay was conducted using the Applied Biosystems ViiA^TM^ Real-Time PCR System by Wcgene Biotechnology (China). The primers were shown in Table 1.

**TABLE 1:** Primers used in the real-time quantitative PCR.

**Target gene**	**GeneBank accession no.**	**Primer sequence (5′-3′)**	**Product size (bp)**
claudin-1	100625166	AAAACCTTCGCCTTCCAG AAATGGCTTCCCTCCTGT	244
occludin	397236	TGCTAGTCGGGTTCGTTT GGTATTAGGACCTGATTGC	177
ZO1	100736682	GCCTCCTGAGTTTGAT CTAACGCCAGTTTCTAT	219
FATP4	100155567	TTCATCAAGACGGTCAGGCG AGACGGTGGCAGCGAATAAG	121
CD36	733702	CATTGCTGGTGCTGTCATT TGTAAACTTCCGTGCCTGT	161
IL6	399500	GATGCTTCCAATCTGGGTTCA CATTTGTGGTGGGGTTAGGG	219
TGFβ	397078	ACCTGACTGGGGTCTTCCTT AGTTCCCAGGCTAGGGGTTA	428
18s	100037957	TCGTTGGAGATCGGAGTG CAGTTTGGGCTTCATTCG	161

### Western-Blot Assay

20 μg of protein samples was applied to the 10% SDS-page for electrophoretic separation and immunoblotting was conducted. Primary antibodies for western-blot assay were as follow: claudin-1 (1:500, 13050-1-AP, United States), occludin (1:50,000, abcam 167161, United States), ZO1 (1:500, 21773-1-AP, United States), CD36 (1:500, 18836-1-AP, United States), FATP4 (1:500, 11013-1-AP, United States), β-actin (1:5,000, 20536-1-AP, United States), p-ERK1/2 (1:1,000, CST 4370, United States), ERK1/2 (1:1,000, 16443-1-AP, United States), p-PPARγ (1:500, GTX 32242, United States), and PPARγ (1:1,000, 16643-1-AP, United States). Secondary antibody was HRP-conjugated Affinipure goat anti-rabbit IgG (1:2,000, SA00001-2, United States).

### Statistical Analysis

Statistical analysis was performed using GraphPad Prism Software 7.0 (GraphPad, United States). Significant differences between two groups were calculated using the unpaired two-tailed *t*-test. Significant differences between multiple groups were evaluated by one-way ANOVA combined with a Turkey’s test. *P* < 0.05 was regarded as statistically significant. ^*^P < 0.05, ^∗∗^*P* < 0.01, ^∗∗∗^*P* < 0.001.

## Results

### Effects of ETEC Gavage on Weaning Piglets

**FIGURE 1:**
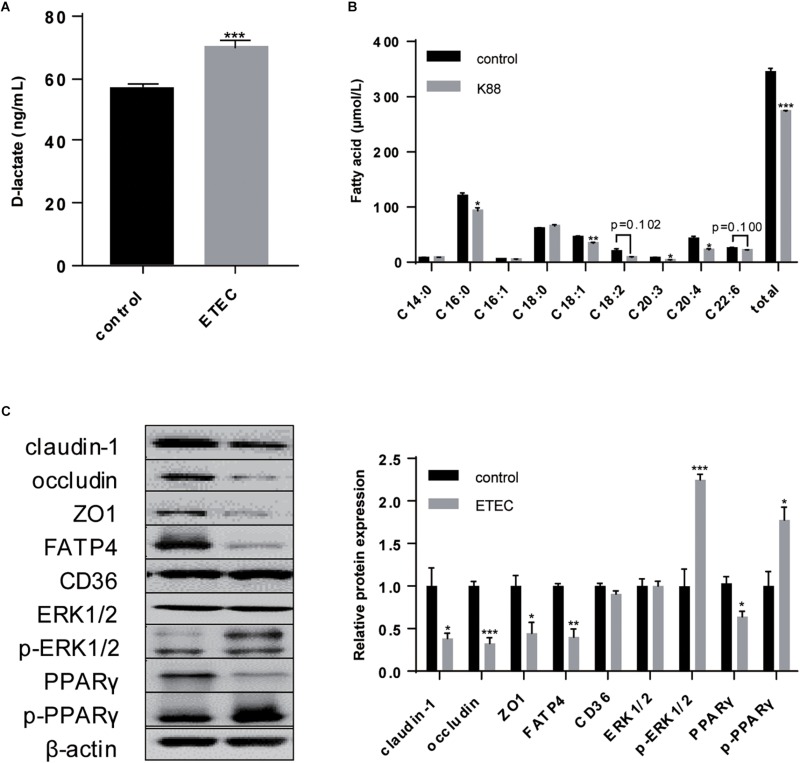
Effects of ETEC gavage on weaning piglets. **(A)** D-lactate content in piglet serum. **(B)** Gas chromatographic analysis of FA in piglet serum 3-h after gavage with olive oil. **(C)** Western-blot analysis of TJs claudin-1, occludin, ZO1; FA uptake associated proteins FATP4 and CD36; signaling pathway proteins ERK1/2, p-ERK1/2, PPARγ, p-PPARγ. Data are presented as mean ± standard error. ^*^P < 0.05, ^∗∗^*P* < 0.01, and ^∗∗∗^*P* < 0.001.

As leak flux diarrhea caused by impaired intestinal barrier is the most symbolic symptom of ETEC infection and a scale to reflect the severity (Adewole et al., 2016), fecal condition was scored consistently within 3-day after gavage (Table 2). On day 8 and day 9, mean fecal score rose to 2 and 2.6, which were simultaneously higher than control and the first day after ETEC gavage (*P* < 0.05). The change in fecal condition indicated 2-day continuous ETEC challenge was capable of inducing typical watery diarrhea within 3-day. Upon ETEC challenge the serum D-lactate rose from 56.645 to 69.917 ng/mL (*P* < 0.001) (Figure 1A), indicating the disruption of intestinal barrier. Altogether these findings implied ETEC infection led to disassembly of intestinal integrity in weaning piglets.

**TABLE 2:** Fecal consistency score^1^ of weaning piglets.

**Treatment**	**Average fecal consistency score: mean ± SD (diarrheal number/total number)^2^**			**Number of piglets dead**
		
	**7 days**	**8 days**	**9 days**	
control	0.5 ± 0.55^a^ (6/6)	0.67 ± 0.52^a^ (6/6)	0.17 ± 0.41^a^ (6/6)	0
ETEC	0.83 ± 1.17^a^ (6/6)	2 ± 0.71^b^ (4/5)	2.6 ± 0.55^b^ (5/5)	1 (on day 12)

*^1^0, normal feces; 1, soft feces; 2, mild diarrhea; 3, severe diarrhea; piglets with a fecal score >1 were considered diarrheal. ^2^Values without a same superscript lowercase differ, *P* < 0.05.*

Combined with the deterioration to intestinal barrier and induction of diarrhea, the uptake ability for C16:0 (*P* < 0.05), C18:1 (*P* < 0.01), C20:3 (*P* < 0.05), C20:4 (*P* < 0.05), and the total (*P* < 0.001) exhibited decrease. In comparison, the concentration of C14:0, whose chain length falls between LCFA and MCFA, exhibited no difference upon ETEC K88 challenge (Figure 1B). As planned we detected the expression of FATP4 and CD36, but only FATP4 showed significant change in protein expression (*P* < 0.05), indicating the tighter association between FATP4 and LCFA uptake in epithelial enterocytes (Figure 1C).

Furthermore, ETEC challenge upregulated the expression of p-ERK1/2 (*P* < 0.001) and p-PPARγ (*P* < 0.05), downregulated the expression of PPARγ (*P* < 0.05), with no significant effects on ERK1/2 (*P* > 0.05). It demonstrated that ETEC initiated phosphorylation of ERK1/2, and promoted the transition of PPARγ into p-PPARγ (Figure 1C).

### Polarization of IPEC-J2 Cell Line

**FIGURE 2:**
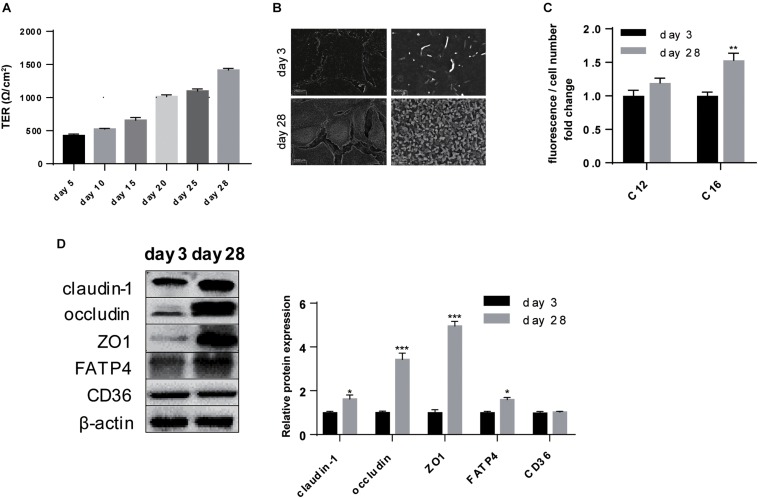
Polarization of IPEC-J2 cell line promoted intestinal barrier and LCFA uptake ability. **(A)** Time-dependent TER measurement of IPEC-J2 cells growing in Transwells. **(B)** SEM visualization of cells grown on day 3 and day 28. Scale bars, 2,500 μm (left) and 20,000 μm (right). **(C)** Uptake of Bodipy-labeled C12:0 and C16:0 normalized by cell counting. **(D)** Western-blot analysis of TJs claudin-1, occludin, ZO1; FA uptake associated proteins FATP4 and CD36. Data are presented as mean ± standard error. ^*^P < 0.05, ^∗∗^*P* < 0.01, and ^∗∗∗^*P* < 0.001.

As enhanced intestinal barrier is a remarkable symbol of a single confluent monolayer constituted by polarized cells, TER was constantly recorded for 28-day until it was stably above 1,000 Ω/cm^2^ which was generally regarded as the marker of successful polarization (Figure 2A). Elevation in intestinal integrity was resulted from promoted protein expression of claudin-1 (*P* < 0.05), occluding (*P* < 0.001), and ZO1 (*P* < 0.001) (Figure 2D). Concurrently, microvilli were obviously more intensive on the surface of plasma membrane after polarization (Figure 2B). These results supported that in our study 28-day was sufficient for polarization and this period was able to be applied for other treatments where Transwell was not suitable.

With regard to the effects of polarization on FA uptake, the uptake measurement showed polarization could promote uptake ability of C16:0 (*P* < 0.01) without any effects on C12:0 uptake (*P* > 0.05) (Figure 2C). As the two possible undertakers of FA uptake in intestinal enterocytes, only FATP4 increasingly expressed (*P* < 0.05) while the protein expression of CD36 showed no difference (*P* > 0.05) (Figure 2D).

### ETEC Challenge Caused Intestinal Barrier Damage on Polarized IPEC-J2 Cell Line

**FIGURE 3:**
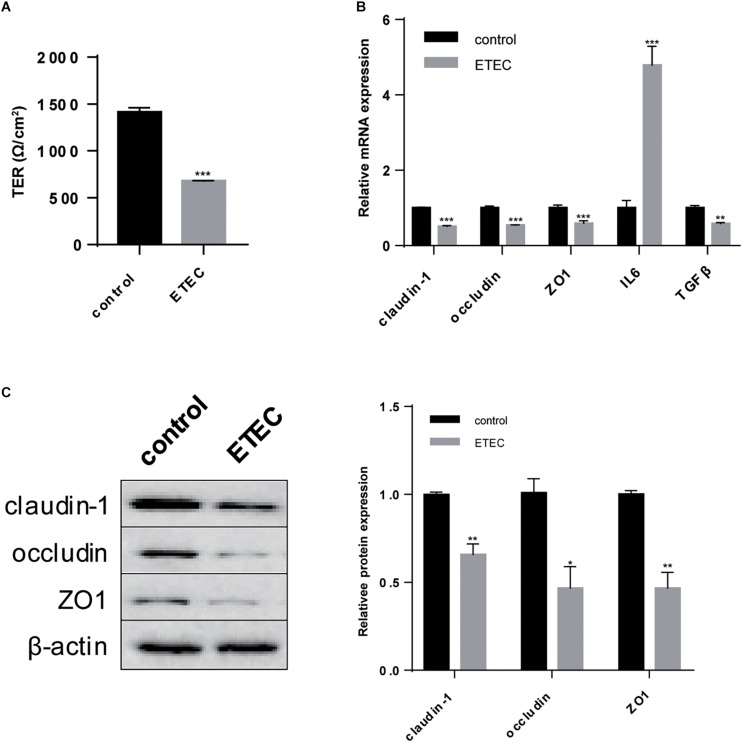
ETEC challenge paralyzed intestinal barrier of polarized IPEC-J2 cell line. **(A)** TER measurement. **(B)** RT-qPCR analysis of TJs claudin-1, occludin, and ZO1; proinflammatory factor IL6 and inflammatory cytokine TGFβ. **(C)** Western-blot analysis of TJs claudin-1, occludin, ZO1. Data are presented as mean ± standard error. ^*^P < 0.05, ^∗∗^*P* < 0.01, and ^∗∗∗^*P* < 0.001.

*Enterotoxigenic E. coli* resulted in leak of intestinal barrier which was reflected in *in vitro* study by decreased TER (*P* < 0.001) (Figure 3A). RT-qPCR analysis showed that transcription of claudin-1, occludin, and ZO1 was all inhibited (*P* < 0.001) (Figure 3B). Western-blot analysis further revealed the lower expression of claudin-1 (*P* < 0.01), occludin (*P* < 0.05), and ZO-1 (*P* < 0.01) (Figure 3C). ETEC infection often combines with intestinal inflammation, thus we also detected the transcription of inflammation-related factors and found an increase in proinflammatory factor IL-6 (*P* < 0.001) with a decrease in inflammatory cytokine TGF-β (*P* < 0.01) (Figure 3B). These data demonstrated ETEC challenge caused intestinal barrier damage and meanwhile inflammatory responses on polarized IPEC-J2 cell line.

### ETEC Induced Disorder in LCFA Uptake of Polarized IPEC-J2 Cell Line

**FIGURE 4:**
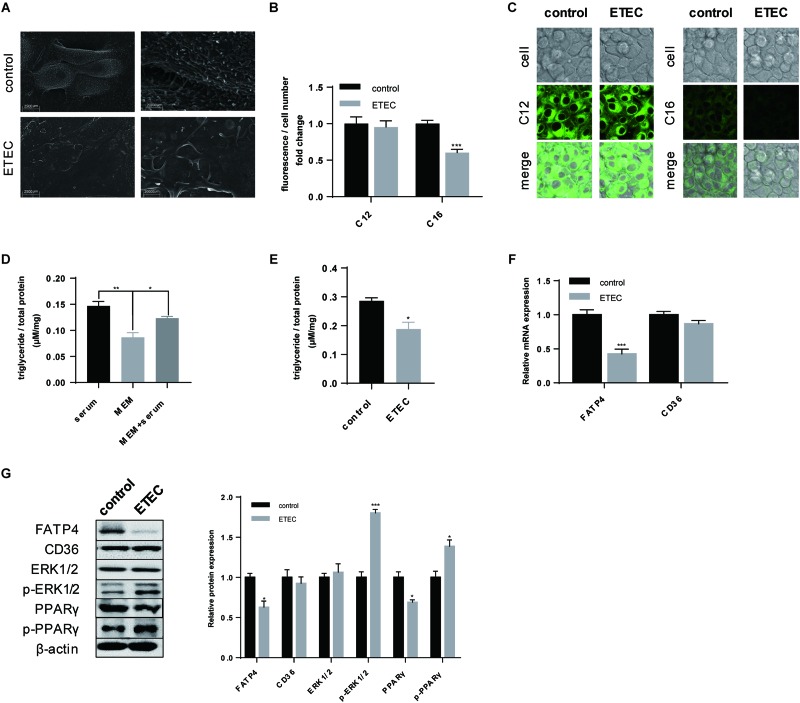
ETEC challenge compromised LCFA uptake and initiated phosphorylation of ERK1/2 and PPARγ. **(A)** SEM visualization. Scale bars, 2,500 μm (left) and 20,000 μm (right). **(B)** Uptake of Bodipy-labeled C12:0 and C16:0 normalized by cell counting. **(C)** Confocal microscopy of Bodipy-labeled C12:0 and C16:0 in IPEC-J2 cells. Scale bars, 10 μm. **(D)** Intracellular TG accumulation measurement of consistent 3.5-h cultivation in MEM + 10% FBS (serum), 3.5-h cultivation in pure MEM (MEM), and 3-h starvation in MEM followed by 0.5-h cultivation in MEM + 10% FBS (MEM + serum). **(E)** The control and ETEC group were treated the same as MEM + serum whereas cells in ETEC group were firstly cultivated in ETEC-containing MEM. **(F)** RT-qPCR analysis of FATP4 and CD36. **(G)** Western-blot analysis of FA uptake associated proteins FATP4 and CD36; signaling pathway proteins ERK1/2, p-ERK1/2, PPARγ, p-PPARγ. Data are presented as mean ± standard error. ^*^P < 0.05, ^∗∗^*P* < 0.01, and ^∗∗∗^*P* < 0.001.

In the next we focused on the effects of ETEC on LCFA uptake of polarized IPEC-J2 cells. After ETEC infection the intensive microvilli on plasma membrane of polarized cells substantially shed off (Figure 4A). Quantitative analysis of Bodipy-labeled FA showed ETEC challenge reduced LCFA content (*P* < 0.001) but did not influence MCFA content (*P* > 0.05) (Figure 4B). Confocal microscopy not only indicated the different uptake levels of LCFA and MCFA, but also pointed within the same time period more MCFA was transported across the membrane under the same exposure conditions (Figure 4C). Since LCFA transported by epithelial enterocytes was mostly used for TG synthesis, we then detected the TG content to trace the following utilization of LCFA. As shown in the preliminary experiment (Figure 4D), 3-h starvation in MEM was able to exhaust the original TG content (*P* < 0.001) by which excluded the disturbance of basal TG content. When cultivated in MEM containing 10% FBS for 30-min, TG content would increase (*P* < 0.01) to the level of serum treatment (*P* > 0.05) (Figure 4D). Using MEM + serum as control, ETEC infection resulted in decrease in the TG content (*P* < 0.05), by which confirmed the uptake of raw material LCFA for TG synthesis was exacerbated by ETEC (Figure 4E). As for FA uptake associated proteins, merely FATP4 showed reduction in both transcription (*P* < 0.001) (Figure 4F) and translation (*P* < 0.05) (Figure 4G). Besides, upon ETEC challenge, phosphorylation of ERK1/2 (*P* < 0.001) and PPARγ (*P* < 0.05) was initiated at the expense of decreasing in non-phosphorylated PPARγ (*P* > 0.05) (Figure 4G). These data from cellular experiment in barrier damage, FA uptake, and expression of targeted genes were mostly in compatibility with those from ETEC-challenged piglets.

### FATP4 Undertook LCFA Uptake in Polarized IPEC-J2 Cell Line

**FIGURE 5:**
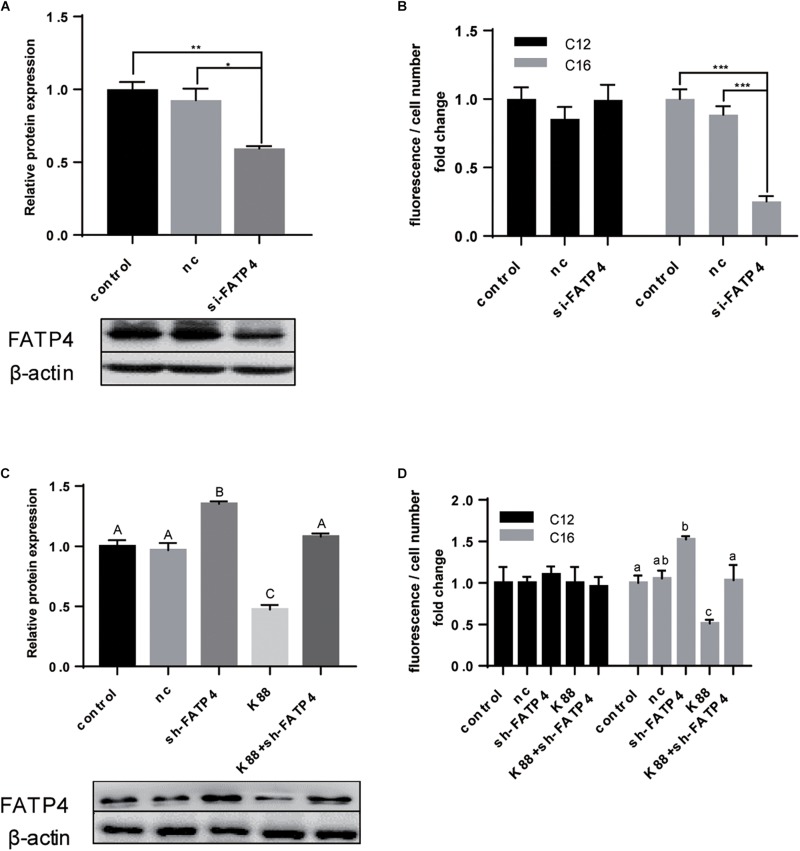
FATP4 was dispensable in LCFA uptake of polarized IPEC-J2 cell line. **(A)** Western-blot analysis of FATP4 transfected with scrambled siRNA (nc) or si-FATP4 (si-FATP4). **(B)** Uptake of Bodipy-labeled C12:0 and C16:0 normalized by cell counting. **(C)** Western-blot analysis of FATP4 transfected with nc, sh-FATP4, and ETEC + sh-FATP4. **(D)** Uptake of Bodipy-labeled C12:0 and C16:0 normalized by cell counting. Data are presented as mean ± standard error. In **(A,B)** ^*^P < 0.05, ^∗∗^*P* < 0.01, ^∗∗∗^*P* < 0.001; in **(C)** values without a same superscript majuscule differ, *P* < 0.01; in **(D)** values without a same superscript lowercase differ, *P* < 0.05.

According to previous results, FATP4, not CD36, showed closer relationship with LCFA uptake either upon ETEC challenge or during the polarization period. We then tried to prove that FATP4 undertook the transmembrane transportation of LCFA from intestinal lumen into enterocytes. After knocking down FATP4 gene by siRNA (Figure 5A), the expression fell by 0.4-fold (*P* < 0.01). Correspondingly, C16:0 content decreased by 0.7-fold (*P* < 0.001) and C12:0 content showed no difference (*P* > 0.05) (Figure 5B). Furthermore, FATP4 gene was overexpressed in cells followed by increased protein expression of FATP4 by 0.35-fold (*P* < 0.01) (Figure 5C). Even after ETEC challenge the expression was still insignificant compared with the control (*P* > 0.05) and was 0.7-fold higher than that of challenged cells without overexpression of FATP4 (*P* < 0.01) (Figure 5C). The uptake of LCFA was in accordance with the increased or decreased expression of FATP4 (Figure 5D). Herein, FATP4 should play a dominating role in LCFA uptake of epithelial enterocytes.

### ERK1/2-PPARγ Worked as the Regulating Pathway of FATP4

**FIGURE 6:**
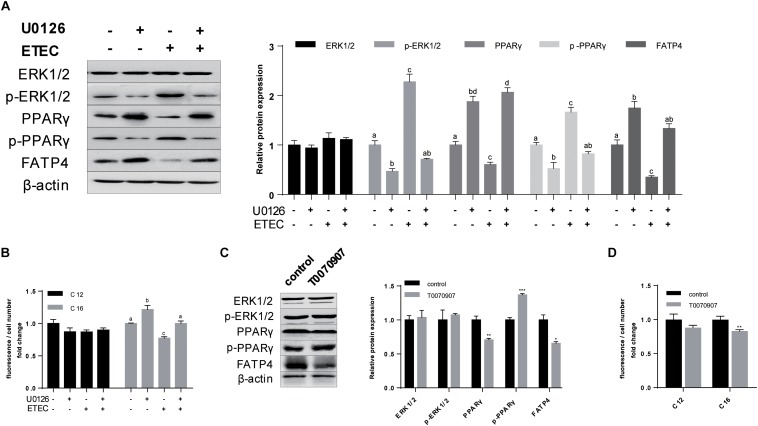
Phosphorylation of ERK1/2 and PPARγ downregulated the expression of FATP4 by which deteriorated LCFA uptake. **(A)** Western-blot analysis of ERK1/2, p-ERK1/2, PPARγ, p-PPARγ, and FATP4 treated with U0126 (+/−) and ETEC (+/−). **(B)** Uptake of Bodipy-labeled C12:0 and C16:0 normalized by cell counting. **(C)** Western-blot analysis of ERK1/2, p-ERK1/2, PPARγ, p-PPARγ, and FATP4 treated with T0070907 (+/−). **(D)** Uptake of Bodipy-labeled C12:0 and C16:0 normalized by cell counting. Data are presented as mean ± standard error. In **(A,B)** values without a same superscript lowercase differ, *P* < 0.05; in **(C,D)** ^*^P < 0.05, ^∗∗^*P* < 0.01, and ^∗∗∗^*P* < 0.001.

In the former results we observed phosphorylation of ERK1/2 and the combining transition of PPARγ into phosphorylated state (Figures 1C, 4G) whereas the mutual relationship was uncertain. Hence, in the first ERK1/2 phosphorylation inhibitor U0126 was applied in polarized cells (Figure 6A). U0126 could inhibited the phosphorylation of ERK1/2 (*P* < 0.05) and PPARγ (*P* < 0.05). The expression of FATP4 (*P* < 0.05) and PPARγ (*P* < 0.05) were both enhanced when treated with U0126. Upon ETEC challenge, U0126 could resist the phosphorylation and protect FATP4 from disassembly. Bodipy-labeled FA transport assay showed U0126 could promote C16:0 uptake in normal cultivation condition and in ETEC K88 challenge (Figure 6B).

To further investigate the relationship between ERK1/2 and PPARγ, PPARγ antagonist T0070907 was used. Western-blot analysis indicated T0070907 could promote p-PPARγ (*P* < 0.001) and simultaneously inhibited expression of PPARγ (*P* < 0.01) and FATP4 (*P* < 0.05), while no effects on ERK1/2 and its phosphorylated state were observed (*P* > 0.05) (Figure 6C). Bodipy-labeled FA uptake assay revealed T0070907 could hinder C16:0 uptake (*P* < 0.01) (Figure 6D). These data indicated that cascaded phosphorylation of ERK1/2 and PPARγ by ETEC K88 could inhibit the expression of FATP4 by which interfered LCFA uptake.

## Discussion

The current study focuses on the effects and the underlying mechanism of ETEC challenge on FA uptake from intestinal lumen. For this purpose, we firstly built ETEC K88-induced intestinal damage models on weaning piglets and polarized IPEC-J2 cell line. Then we analyzed the expression of associated factors combined with the uptake ability for LCFA and MCFA as an exploration for their roles in FA uptake. The classification of LCFA and MCFA is due to Beermann et al. (2003).

Here we exhibit ETEC would cause dysfunction in the uptake of LCFA, not MCFA or FA with approximate chain length, through interference of FATP4 expression. The crucial role of FATP4 in LCFA uptake is further supported by knock-down and overexpression assays together with FA uptake analyses. These results are in accordance with classical theories that LCFA uptake is positively related to the expression of FATP4 (Stahl et al., 1999; Buttet et al., 2014). In addition, within the same period, the Bodipy fluorescence intensity of C12:0 is obviously brighter than that of C16:0 whether it is upon ETEC challenge or not. It indicates that transmembrane transport of MCFA from intestinal lumen should be faster than LCFA and is independent of FATP4. As far as we are concerned, FA uptake mode is mainly affected by water solubility (Hamilton, 1998) which is determined by chain length (Alsabeeh et al., 2018). With longer the chain length is, FA hydrophobicity will coefficiently increase and consequently enhance the transport difficulty. According to PubChem database^1^, the water solubility of C16:0 (0.04 mg/L, 25°C) is greatly lower than that of C14:0 (1.07 mg/L, 30°C) and C12:0 (1.07 mg/L, 25°C). Though acidity may have an effect on the transport efficiency, supplementation of buffer solution in working solutions is capable of leveling up the difference in pH values, demonstrating the chain length and the corresponding water solubility play the dominating role in FA uptake mode. The conventional theory about rapid utilization of MCFA usually attributes that they do not necessarily require the esterification process to reform into TG and are able to directly join into the portal vein (Odle, 1997). Our study lends support that MCFA transmembrane transport from intestinal lumen may also be unlike from that of LCFA and in a mode of FATP4-independent diffusion.

On the contrary to our prediction, CD36 is not affected in weaning piglets or cells challenged by ETEC and thereby has a relatively indirect association with LCFA uptake. CD36 is reported to exist in adipocytes, myocytes, and enterocytes, where CD36 on the one hand transports FA from endosome to cytoplasma, and on the other hand, assists to recruit FA to plasma membrane by which improves the transport efficiency (Glatz and Luiken, 2017). Notably, these studies are mostly based on albumin binding FAs existing in bloodstream (Storch and Corsico, 2008; Niot et al., 2009; Siddiqi et al., 2010; Lagakos et al., 2013; Nakamura et al., 2014). In our study, nevertheless, FA bundles into micelles with the assistance of bile acid or an analog taurocholic acid sodium salt, and then enters into intestinal enterocytes independent of transportation of CD36. Even so, we cannot rule out the contribution of CD36 to FA recruitment during the uptake process.

*Enterotoxigenic E. coli* challenge on weaning piglets initiates the cascaded phosphorylation of ERK1/2 and PPARγ in jejunum, which in turn decreases the expression of non-phosphorylated PPARγ. To unveil their crosstalk, ERK1/2 inhibitor U0126 and PPARγ antagonist T0070907 were applied to the polarized cells, respectively, indicating inhibitory phosphorylation of ERK1/2 will impede the transition of PPARγ from activated state (non-phosphorylated) to inactivated state (phosphorylated) (Kaplan et al., 2010). Phosphorylation of PPARγ by phosphorylated ERK1/2 interferes PPARγ’s binding to the peroxisome proliferator response element in the promoter region of targeted genes (Burgermeister and Seger, 2007, 2008; Kaplan et al., 2010; Moon et al., 2010). The expression of non-phosphorylated PPARγ is thus related to FATP4 expression and LCFA uptake. Moreover, the deteriorative effects of ETEC on LCFA uptake can also be alleviated by U0126, verifying the disturbing role of signaling pathway ERK1/2-PPARγ in FATP4 protein expression and LCFA uptake as well.

For *in vitro* assay polarization of IPEC-J2 cell line is of great importance in uptake research especially to this study. Transporting system is tightly correlative with cell polarity as a result of differentiation in cell morphology, functions and correspondingly the expression of related genes (Altschuler et al., 2008). Therefore, to better imitate the physiological condition of intestinal lumen, IPEC-J2 cell line is necessarily polarized by serum-free cultivation in 0.4 μm Transwell. As shown in our study polarization can intensify the microvilli, which is a remarkable appearance of polarized IPEC-J2 cells (Schierack et al., 2006; Geens and Niewold, 2011), and promote the uptake ability for LCFA possibly due to the accumulation of FATP4 in the same site (Stahl et al., 1999). Moreover polarization strengthens cellular barrier by induction in TJ expression (claudin-1, occludin and ZO1) compared with unpolarized cells. Interestingly at the very beginning when we exposed unpolarized cells to bacterial challenge, only TJ expression showed reduction but neither FATP4 nor CD36 was affected, accompanied with unaffected LCFA and MCFA uptake even if we increased the bacteria intensity to 2-fold (data not shown). Herein, we speculate that the basal line of FATP4 expression or FA uptake in unpolarized IPEC-J2 cell line is too low to respond to any bacterial stimuli.

In the field of neonatal nutritional research, the conventional “rodents – Caco-2” model has showed a series of limitations in simulating the human intestinal conditions thus we utilized “pig – IPEC-J2” model instead (Stremmel et al., 2017). For decades rodents (mice, rats) were used in nutritional research due to the low costs for acquisition and maintenance, uniformly standardized environment, highly ethical acceptance, and the rapid growth rate (Renner et al., 2016). However, the tremendous divergences in growth rate, body size, cell divisions and intestinal morphological structure determine rodents may not be appropriate for neonatal nutritional studies (Rangarajan and Weinberg, 2003; Nguyen et al., 2015). Moreover, some enteric diseases occurring in human beings cannot be successfully manifested in mice or rats (Jeong et al., 2010). In the contrast pigs share closer similarities in lifespan, dietary habits, intestinal morphology and gastrointestinal microbiota with human beings (Patterson et al., 2008; Gonzalez et al., 2015; Roura et al., 2016). Porcine genome mapping shows the pig genome is only 7% smaller than humans whereas the rodent genome is about 14% smaller than human beings (Gonzalez et al., 2015). Based on the above homologies piglets have been applied widely in early-life nutrition and in lipid metabolism research (Litten-Brown et al., 2010; Mudd and Dilger, 2017). With regard to *in vitro* study, Caco-2 cell line is the classical model both for small intestine and for large intestine research. Even if it is originated from colon, after polarization Caco-2 can differentiate brush-like microvilli (Sambuy et al., 2005) and exhibit the active transport system just like the small intestine (Rousset, 1986). However, FATP4 does not express in colon site *in vivo* (Stahl et al., 1999). In addition, the activity of monoacylglycerol transferase in polarized Caco-2 cell line is lower than the that of normal condition in the small intestine (Trotter and Storch, 1993), which may possibly result in the decreased ability of synthesis and secretion of chylomicrons (Luchoomun et al., 1997). As a comparison, IPEC-J2 is a cell line directly originated from neonatal piglet jejunum and has an instinct advantage in mimicking the condition of small intestine. Due to the morphological and functional similarities between pig and human, it has an innate advantage on mimicking early-life intestinal conditions (Brosnahan and Brown, 2012). Currently this cell line is increasingly being used in microbial infection studies including ETEC K88. Considering the facts, in this study we selectively employed weaning piglets and polarized IPEC-J2 cell line to mimic the situation of small intestine of weaning mammals including human beings. In the present study these two models show outstanding compatibility in the intestinal permeability, protein expression (TJs, FA uptake associated proteins, and signaling proteins) and FA uptake change upon ETEC challenge, exhibiting the reliability of “pig – IPEC-J2” in the intestinal research.

Apart from the current results, the future work will be focused on the aspects as follow: (1) In order to mimic the infection situation happened on weaning mammals, only whole bacteria were used in this study. But which element of ETEC on earth initiates the phosphorylation of ERK1/2-PPARγ is not known yet. (2) According to our study, the unaffected uptake of MCFA upon ETEC challenge demonstrates there should be a different mode of MCFA transmembrane transport from the lumen side. Whether it requires the involvement of other proteins or is just diffusion relies on more in-depth research in the molecular level.

## Conclusion

In conclusion, the *in vivo* and *in vitro* results from weaning piglets and IPEC-J2 cell line indicates ETEC challenge will cause dysfunction in FATP4-dependent LCFA uptake in small intestine. ETEC can phosphorylate ERK1/2 and consequently transit PPARγ (activated) into p-PPARγ (inactivated) by which downregulates the expression of FATP4 as well as LCFA uptake.

## Data Availability

All datasets generated for this study are included in the manuscript and/or the supplementary files.

## Ethics Statement

The Zhejiang University Animal Care and Use Committee.

## Author Contributions

ZL and HL designed the study and carried out the animal and cell experiments. ZL analyzed the data and organized the manuscript. BX edited the figures. YW supervised the whole study and corrected the final version of the manuscript.

## Conflict of Interest Statement

The authors declare that the research was conducted in the absence of any commercial or financial relationships that could be construed as a potential conflict of interest.

## Footnotes

1 https://pubchem.ncbi.nlm.nih.gov/

## Abbreviations

CD36: cluster of differentiation 36
CFU: colony-forming unit
ERK: extracellular signal-regulated kinase
ETEC: *Enterotoxigenic Escherichia coli*
FA: fatty acid
FATP: fatty acid transporter
FBS: fetal bovine serum IL6, interleukin 6 LCFA, long-chain fatty acid
MCFA: medium-chain fatty acid
PPARγ: peroxisome proliferator-activated receptor gamma
RPM: revolutions per minute
RT-qPCR: real-time quantitative polymerase chain reaction
SEM: scanning electron microscopy
TER: transepithelial electrical resistance
TG: triglyceride
TGFβ: transformed growth factor β
TJ: tight junction
ZO1: zonula occluden 1.
